# Arginine Delays Postharvest Softening of Button Mushroom (*Agaricus bisporus*) via Lipid Metabolism Regulation

**DOI:** 10.3390/foods14244359

**Published:** 2025-12-18

**Authors:** Dandan Xu, Lu Gao, Xiaoyan Mu, Tan Wang, Junsong Liang, Qi Wang, Qiuhong Niu

**Affiliations:** 1 College of Life Science, Nanyang Normal University, Nanyang 473061, China; happyxudandan@126.com (D.X.);; 2 Department of Plant Pathology, College of Plant Protection, China Agricultural University, Beijing 100193, China

**Keywords:** MDA, metabolomics, transcriptomics, unsaturated fatty acids

## Abstract

Postharvest storage and quality maintenance represent significant constrains for the marketability and long-distance exportation of button mushroom (*Agaricus bisporus*). Protective techniques such as arginine application has been demonstrated to extend the shelf life of button mushroom. However, the underlying mechanism by which arginine mitigates postharvest softening in button mushroom require further elucidation. In this study, comprehensive physiology, metabolomics and transcriptomics analyses of button mushroom following arginine treatment were conducted to investigate its potential mechanisms of action. Physiological analysis showed that arginine treatment (1.5 g L^−1^) markedly alleviated the postharvest softening of button mushroom, resulting in a 23.8% increase in firmness, reduced malondialdehyde (MDA) content, suppressed activities of phenylalanine ammonia-lyase (PAL) and polyphenol oxidase (PPO), and maintained elevated superoxide dismutase (SOD) activity. Integrated transcriptomic and metabolomic analyses demonstrated that arginine application significantly altered lipid-related metabolites, including free fatty acids, lysophosphatidylcholine (LPC), lysophosphatidylethanolamine (LPE) and phosphatidylcholine (PC). Notably, arginine treatment increased the levels of unsaturated fatty acids (UFAs). Transcriptomic analysis further revealed that differentially expressed genes (DEGs) were predominantly enriched in lipid metabolism pathways following arginine treatment. Specifically, arginine application stimulated the lipid metabolism by upregulating genes associated with fatty acid desaturation (FAD), while downregulating genes related to phospholipases A2 (PLA2). These findings collectively demonstrate that arginine effectively mitigates postharvest softening of button mushroom by modulating lipid metabolism.

## 1. Introduction

Button mushroom (*Agaricus bisporus*) is an edible fungus species that has a pleasant but subtle umami taste that blends earthy and meat-like flavors and is found all over the world [1]. China is the largest producer and most potent exporter of button mushroom in the world, whose production ranging in the fourth sequence of the production line with about 3.35 million tons per year [2]. However, the shelf life of button mushroom is limited to 3–4 d at room temperature because of a series of physiological and biochemical changes, such as browning, water loss, senescence, and microbial attack, which have already become the most important constraint in enlarging the market and the long-distance exportation of button mushrooms [3]. In this respect, special protection techniques are required to maintain their quality and extend the shelf life.

There are various treatments for maintaining the quality of mushrooms, including application with essential oils, ergothioneine, methyl jasmonate, UV-B, and power ultrasound [4,5,6,7,8]. Some amino acids are traditionally applied to enhance postharvest preservation as a natural and eco-friendly antifungal compound and provide products with a sheen appearance and make them more attractive to consumers [9]. Arginine represents an emerging eco-friendly postharvest technology that aligns with the growing demand for sustainable preservation strategies. Similar to bio-based edible coatings that maintain membrane integrity and antioxidant activity in fruits, arginine offers a natural alternative to synthetic preservatives [10]. Arginine is one of the most functionally diverse amino acids and serves as a biosynthetic precursor of polyamine, agmatine, proline, and nitric oxide, which are important messenger molecules in most physiological and biochemical processes, growth and development, and stress resistance [11]. Recently, Xue et al., found that arginine treatment provided a stable energy supply for broccoli to extend storage time [12]. Chang et al., demonstrated that arginine induces the resistance of postharvest jujube fruit against *Alternaria* rot by reducing H_2_O_2_ content and promoting the phenylpropane metabolism [13]. Mahmoudi et al., reported that new coating treatments based on arginine and chitosan applied to plum fruit ameliorate chilling injury during cold storage by enhancing ROS scavenging system activity [14]. Thus, arginine could prime tolerance and might be a promising strategy for attenuating postharvest decay and enhancing defense response against fungal pathogens and chilling injury. Furthermore, arginine was discovered to delay postharvest ripening of tomato fruit by triggering the ethylene biosynthesis [15]. Shu et al., reported that L-arginine treatment attenuated postharvest decay of strawberry by promoting the nitric oxide synthase pathway [16].

The cellular membrane is a complex and dynamic structure that is essential for the biological function of cells [17]. As the main component of the cellular membrane, lipid metabolism plays a pivotal role in maintaining membrane integrity through the coordinated actions of key enzymes. Alterations in its physicochemical properties can disrupt metabolic homeostasis, triggering a cascade of biochemical reactions that may ultimately lead to cellular damage and tissue deterioration [18]. Huang et al., showed that membrane lipid metabolism in Hami melon was alleviated by *n*-butanol treatment and thus mitigating chilling injury [19]. Cao et al., found that melatonin reduces postharvest decay of blueberries during storage by regulating membrane lipid metabolism [20]. The modulation of lipid metabolism may contribute to the reduction in postharvest senescence. In button mushrooms, arginine has been proven to have a significant effect on prolonging the shelf life of white button mushrooms [21].

While physiological advances have been made in understanding arginine-regulated postharvest senescence, the intricate regulatory mechanisms of lipid metabolism, particularly the specific roles of key genes and metabolites, require comprehensive elucidation and further investigation. This study aims to investigate the impact of arginine application on postharvest button mushrooms, and metabolomic and transcriptomic analyses were applied to unravel the regulatory effects of arginine treatment on membrane lipid metabolism in postharvest button mushrooms. By exploring the changes in gene expression and metabolite profiles related to lipid metabolism, this research provides novel insights into arginine application strategies for the storage quality preservation of edible mushrooms.

## 2. Materials and Methods

### 2.1. Mushroom and Preharvest Arginine Treatment

Mushrooms were harvested at a uniform developmental stage and selected based on consistent size, absence of visible damage, and lack of fungal infection. Arginine was purchased from J&K Scientific Ltd. (Beijing, China), and stock solutions were prepared in sterile water. Preharvest arginine treatment experiments were conducted at a mushroom farm in Nanyang, China. The growing fruiting bodies of button mushrooms were sprayed with arginine solution at different concentrations (0.5, 1.5, and 4.5 g L^−1^) and deionized water (control group) before 24 h of harvest time. After 24 h, fruiting bodies of button mushroom (cap diameter approximately 3–4 cm) with uniform size and without any visible damage or fungal infection were harvested and selected for the experiment. For each treatment, 90 mushrooms were treated and contained three replicates. All treated mushrooms were stored in plastic bags and kept in a refrigerator (4 ± 0.5 °C). The cap browning and firmness were measured every two days. For firmness evaluation, a penetration test was conducted with a texture analyzer (TA.XTC-16, BosinTech, Shanghai, China) equipped with a 2 mm diameter probe (Speed 10 mm s^−1^, depth 5 mm) on 30 mushrooms for each treatment [22]. For arginine treatment, selected mushrooms were air-dried at 20 °C for 30 min.

### 2.2. Sample Preparation

Based on preliminary experiments showing that 1.5 g L^−1^ arginine exhibited marked efficacy on delaying postharvest senescence of mushrooms, fruiting bodies treated with arginine at 1.5 g L^−1^ or deionized water were collected for further analysis. Representative time of 12 h and 48 h (according to the RT-qPCR results in pre-experiment) after treatment were selected for transcriptomic and metabolomic analyses. The samples of the arginine group collected at 12 h and 48 h were designated as Arg-12h and Arg-48h, and the control group at 12 h and 48 h were designated as CK-12h and CK-48h, respectively. Ten mushrooms per replicate of each treatment were taken immediately after 12 h and 48 h of storage at 4 °C, immediately frozen in liquid nitrogen, and then stored at −80 °C for future analysis.

### 2.3. Determination of Enzyme Activities and Malondialdehyde (MDA) Content

Enzymatic activity and MDA (malondialdehyde) content of mushrooms were measured according to a previous method with some modifications [23]. The activities of superoxide dismutase (SOD), phenylalanine ammonia lyase (PAL), and polyphenol oxidase (PPO) in mushrooms were measured using commercially available detection kits (Nanjing Jiancheng Bioengineering Institute, Nanjing, China). One unit of SOD, PAL, and PPO activity was expressed as U g^−1^ fresh weight. For MDA measurement, mushroom sample was grounded with 50 g L^−1^ trichloroacetic acid (TCA) in the ratio of 1:5 (*w*/*v*) and centrifuged for 10 min at 4000× *g*, supernatant was mixed with 0.67% thiobarbituric acid (TBA) in the ratio of 1:1 and incubated for 20 min in boiling water, then cooled quickly and centrifuged for 10 min at 4000× *g*. Absorbance of the supernatant was measured immediately after cooling and centrifugation at 532 nm, and corrected for non-specific turbidity by subtracting the absorbance at 600 nm. MDA concentration was expressed as μmol kg^−1^ fresh weight.

### 2.4. Widely Targeted Metabolomic Analysis

Metabolites were extracted following the methodology reported by Yang et al. and with minor modifications. Briefly, freeze-dried control and arginine-treated mushroom samples were ground, and 50 mg samples were mixed with 1.2 mL of pre-cold 70% methanol, then the extraction mixture was vortexed six times at a frequency of 30 s every 30 min [24]. All samples were centrifuged at 10,500× *g* for 3 min, and the extracts were filtered with a 0.22 μm pore size before UPLC-MS/MS analysis. Quality-control samples and reference standards were included throughout the analysis to ensure reproducibility and reliability of the metabolomic data.

The extracts were injected into a UPLC (ExionLCTM AD, Foster City, CA, USA) equipped with Agilent SB-C18 (1.8 µm, 2.1 mm × 100 mm) and coupled with a MS/MS system (Applied Biosystems 6500 QTRAP, Boston, MA, USA). The mobile phase was 0.1% formic acid in water (phase A) versus 0.1% formic acid in acetonitrile (phase B). The gradient program was 95:5 (phase A/phase B, *v*:*v*) at 0 min, 5:95 (phase A: phase B, *v*:*v*) at 9 min, held for 1 min, 95:5 (phase A/phase B, *v*:*v*) at 11.1 min, held for 2.9 min. Mass spectrometry analysis was performed by Analyst 1.6.3. The metabolites were identified according to the secondary spectral information and the multiple reaction monitoring (MRM) modes using a self-constructed database, MWDB (Metware Database), as a reference. Metabolites were quantified based on the squares-discriminant analysis (OPLS-DA) results, and variable importance in projection (VIP) > 1 and fold change ≥ 1.5 or fold change ≤ 0.67 were considered to be differentially accumulated.

### 2.5. Transcriptomic Analysis

RNA Seq and bioinformatics analysis for control (CK-12h, CK-48h) and arginine-treated (Arg-12h, Arg-48h) mushrooms were conducted according to the report of Mou et al. [25]. Total RNA extracted from three biological replicates of mushrooms was used to generate sequencing libraries using NEBNext^®^ UltraTM RNA Library Prep Kit for Illumina^®^ (NEB, Ipswich, MA, USA). The library quality was assessed on the Agilent Bioanalyzer 2100 system. The gene expression levels were estimated by RSEM and then normalized into Fragments Per Kilobase of transcript per Million fragments mapped (FPKM) based on the gene length. Differentially expressed genes (DEGs) were screened by applying DESeq2 with a threshold of the log2|FC| ≥ 1 and FDR < 0.05. The KEGG and GO enrichment analyses for all DEGs were calculated based on the hypergeometric test.

### 2.6. Weighted Gene Co-Expression Network Analysis (WGCNA)

After discarding no expression genes, weighted gene co-expression network analysis (WGCNA, version 1.73) was completed on the DEGs to generate co-expression networks in R software (version 3.0) [26]. The eigengene value for each module was calculated and used to assess its correlation with each physiological parameter of firmness and MDA content in mushrooms. R and R Studio were used to run the WGCNA R package v.1.73.

### 2.7. Quantitative Real-Time PCR (qRT-PCR) Analysis

qRT-PCR analysis was conducted to assess the quality and reproducibility of RNA-Seq data. Total RNA isolation was performed by the Trizol method [27]. The quality of RNA was determined by using a microspectrophotometer (NanoDrop 2000, Thermo Scientific, Waltham, MA, USA), and then, reverse transcription was conducted with a HiScript II 1st Strand cDNA Synthesis Kit (Vazyme, Nanjing, China). Six representative DEGs were selected for qRT-PCR validation, and the expression level was calculated according to the 2^−ΔΔCt^ method. All reactions were performed three times, and the *AbTubulin* gene (AGABI2DRAFT_195658) was used as an internal control for normalization.

### 2.8. Statistical Analysis

All statistical analyses were performed using SPSS Statistics 22.0 (IBM, New York, NY, USA) software. Data from physiological and metabolite analyses were subjected to one-way analysis of variance (ANOVA). Differences at *p* ≤ 0.05 were considered statistically significant, and means were separated by Duncan’s multiple range test. Heat maps of DAMs and DEGs were prepared using the Metware Cloud (https://cloud.metware.cn/, accessed on 15 December 2025).

## 3. Results

### 3.1. Arginine Treatment Retards Postharvest Senescence of Mushrooms

**Figure 1:**
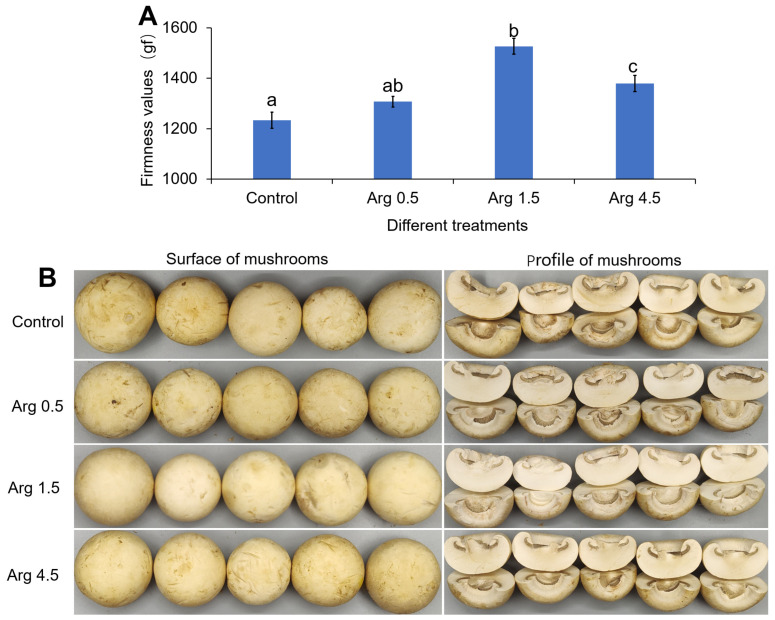
Effect of arginine application (0 as control, 0.5, 1.5, and 4.5 g L^−1^) on firmness and cap browning of mushrooms under refrigeration. (**A**) The firmness of mushrooms during storage at 4 °C for 8 d. (**B**) The cap browning symptoms of mushrooms during storage at 4 °C for 8 d. Each value is expressed as mean ± standard error (n = 3). Different letters indicate significant differences (*p* < 0.05) among treatments.

Color and firmness are important quality parameters and are usually considered as typically reliable indicators of the shelf life potential of button mushrooms. As shown in Figure 1A, the firmness values of mushrooms after treating with arginine were significantly higher than those in the control group, and reached the highest value under the treatment of 1.5 g L^−1^ arginine, with the improvement efficiency reaching 23.8%. Furthermore, the surface and profile of mushrooms in the 1.5 g L^−1^ arginine-treated group exhibited a better appearance and lower browning degree when compared to other treatments (Figure 1B). These results suggest that arginine could markedly delay postharvest decay of button mushrooms during storage.

### 3.2. Changes in Malondialdehyde (MDA) Content and Enzyme Activities

**Figure 2:**
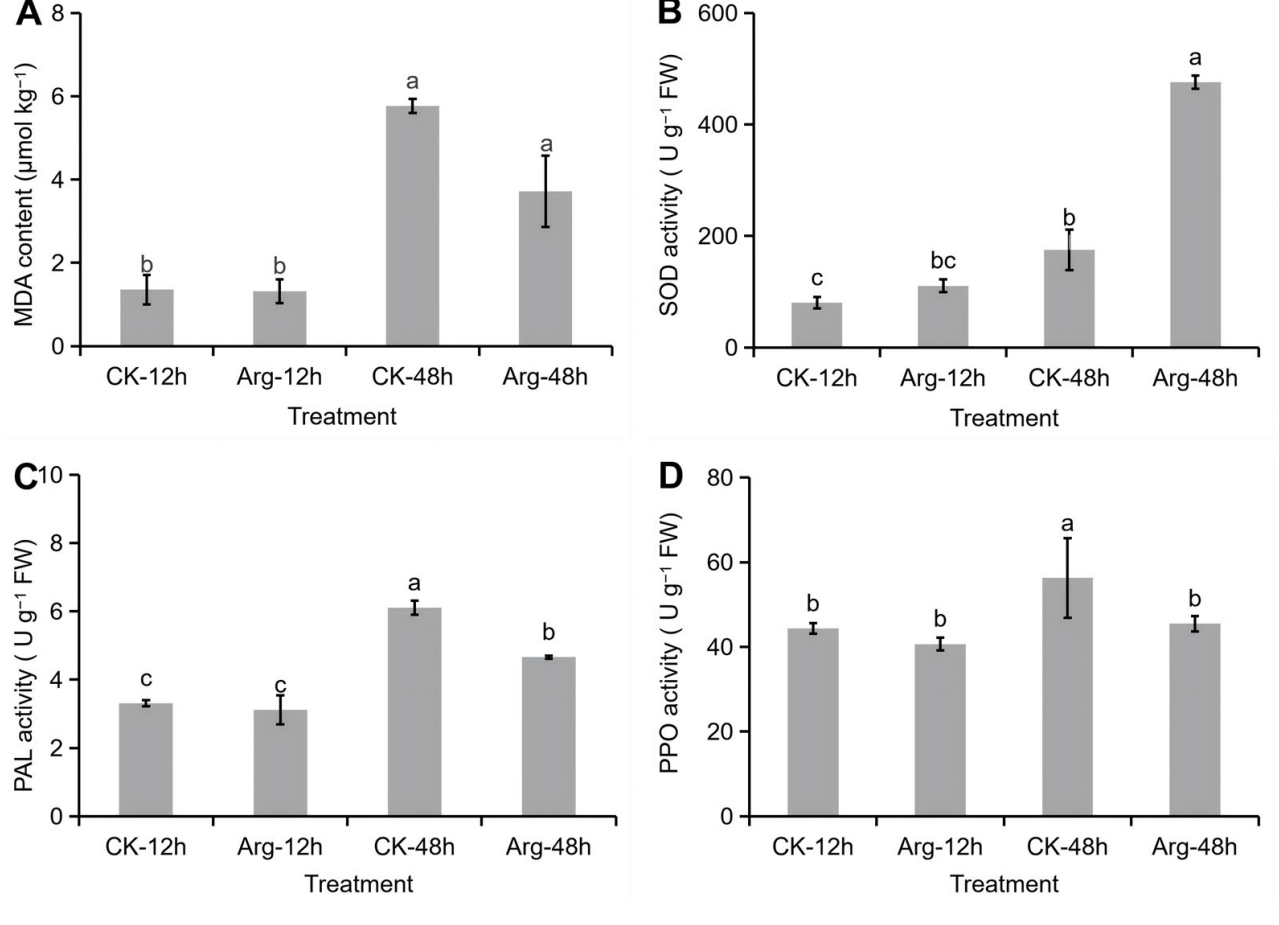
Effect of arginine application on MDA content, SOD activity, PAL activity, and PPO activity of mushrooms under refrigeration. (**A**) MDA content. (**B**) SOD activity. (**C**) PAL activity. (**D**) PPO activity. Each value is expressed as mean ± standard error (n = 3). Different letters indicate significant differences (*p* < 0.05) among treatments.

Malondialdehyde (MDA) is one of the products of membrane lipid peroxidation. As shown in Figure 2A, the content of MDA in mushrooms increased following the extension of storage time. However, the content of MDA in mushrooms under arginine application is lower than that in the control group. The enzymes of SOD, PAL, and PPO play a crucial role in postharvest senescence. As shown in Figure 2B, the SOD activity of mushrooms increased over time and remained at higher levels in the arginine group than in the control group. The trends of PAL and PPO activities were consistent and with a slowing increasing trend over time, and arginine treatment exhibited lower activity than the control group (Figure 2C,D). These results demonstrated that arginine application could decrease MDA content and activities of enzymes PAL and PPO, and maintain a high level of SOD activity in mushrooms during storage. The reduction in MDA content, a product of lipid peroxidation, showed a consistent relationship with the enhanced activities of antioxidant enzymes, particularly SOD. This coordinated response suggests metabolic coherence in the arginine-induced defense mechanism against oxidative stress.

### 3.3. Metabolomic Profiles After Arginine Application

**Figure 3:**
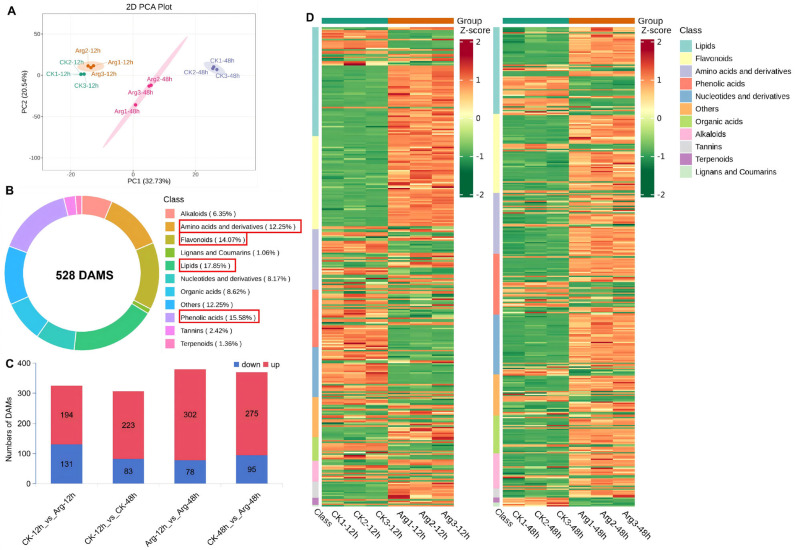
Overview of metabolome analysis in control and arginine treated mushrooms during cold storage. (**A**) PCA of metabolome data. (**B**) Categories and the proportion of each kind of differentially accumulated metabolites (DAMs) in mushrooms. (**C**) The numbers of DAMs in mushrooms. (**D**) Heatmaps of DAMs in Arg-12h vs. CK-12h and Arg-48h vs. CK-48h.

UPLC-MS/MS-based widely targeted metabolomics analysis was conducted to better comprehend metabolite dynamics between the control (CK-12h, CK-48h) and arginine-treated (Arg-12h, Arg-48h) mushrooms. PCA showed that the application of arginine induced remarkable metabolic variations in mushrooms (Figure 3A). The metabolomic profiles of the control group and the arginine-treated group could be separated, and the first two principal components (PC1 and PC2) contributed 32.73% and 20.54% of the variance, respectively. A total of 528 DAMs in mushrooms were detected, belonging to 11 metabolite classes, including lipids (17.85%), phenolic acids (15.58%), flavonoids (14.07%), amino acids and derivatives (12.25%), organic acids (8.62%), nucleotides and derivatives (8.17%), alkaloids (6.35%), tannins (2.42%), terpenoids (1.36%), linins and coumarins (1.06%), and others (12.25%) (Figure 3B). These results indicated that lipids, phenolic acids, and flavonoids are the main categories of DAMs in mushrooms.

Differentially accumulated metabolites (DAMs) were identified in four comparisons: Arg-12h vs. Arg-48h (380 DAMs: 302 up, 78 down), CK-12h vs. CK-48h (306 DAMs: 223 up, 83 down), Arg-48h vs. CK-48h (370 DAMs: 275 up, 95 down), and Arg-12h vs. CK-12h (325 DAMs: 194 up, 131 down) (Figure 3C). Additionally, KEGG enrichment showed that DAMs under control and arginine treatments were mainly enriched in the pathways of lipids and flavonoids, and amino acids and derivatives. lipids metabolism was the most significantly enriched pathway (Figure 3D). Therefore, we speculated that lipids were the main contributors to the DAMs in arginine-delaying mushroom postharvest ripening and senescence.

### 3.4. Differential Accumulation of Lipid Compounds

**Figure 4:**
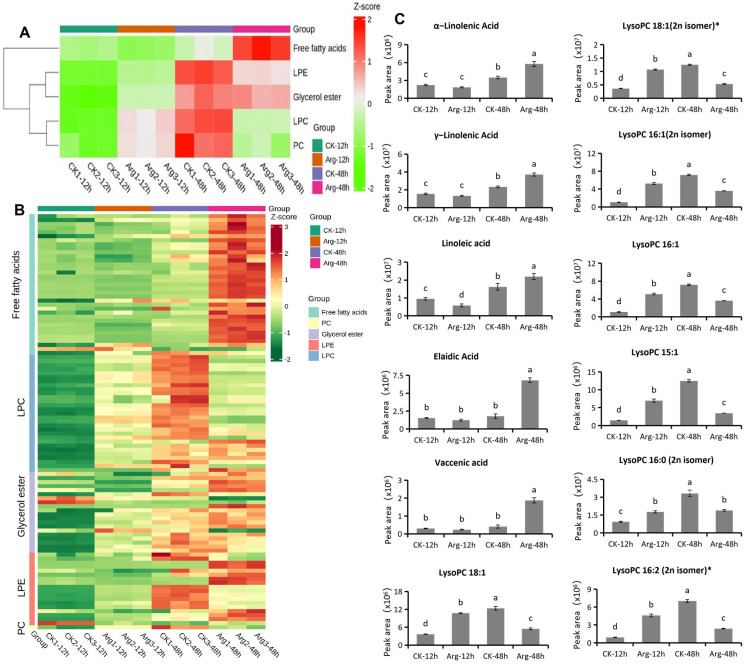
Differential accumulation of lipid compounds in button mushroom. (**A**) The total level of different lipids in different treatments. (**B**) The heatmap of different lipids in comparable groups. (**C**) Different accumulation of five representative unsaturated fatty acids (α-linolenic acid, γ-linolenic acid, linoleic acid, elaidic acid, and vaccenic acid) and seven representative lysophosphatidylcholines (LysoPC 18:1, LysoPC 18:1 (2n isomer)*, LysoPC 16:1 (2n isomer), LysoPC 16:1, LysoPC 15:1, LysoPC 16:0 (2n isomer), and LysoPC 16:2 (2n isomer)*). Each value is expressed as mean ± standard error (n = 3). Different letters indicate significant differences (*p* ≦ 0.05) among treatments.

Analytical results indicated that lipid metabolites were closely related to arginine-delayed postharvest softening of button mushrooms. Thus, we focused on lipids, including free fatty acids, lysophosphatidylcholine (LPC), glycerol ester, lysophosphatidylethanolamine (LPE), and phosphatidylcholine (PC). In total, 103 lipid-related metabolites were identified in control (CK-12h, CK-48h) and arginine-treated (Arg-12h, Arg-48h) mushrooms. Among these metabolites, the level of total free fatty acids in Arg-48h was higher than that in CK-12h, Arg-12h, and CK-48h, whereas the LPC total level in Arg-48h was lower than that in Arg-12h and CK-48h (Figure 4A,B). In the comparison of CK-48h vs. Arg-48h, among 67 lipids, 38 lipids (56.72%) were significantly increased, and with a 4.0-fold difference, three different classes, including free fatty acids, LPE, and glycerol ester, were further categorized, and free fatty acids were abundant in mushrooms (Supplementary Table S1). Moreover, the levels of α-linolenic acid, γ-linolenic acid, linoleic acid, elaidic acid, and vaccenic acid, which belong to unsaturated fatty acids (UFAs), in Arg-48h were significantly higher than those in CK-12h, Arg-12h, and CK-48h (Figure 4C). In addition, the content of seven representative LPCs, namely lysoPCs (C18:1, C18:1 (2n isomer)*, C16:1, C16:1 (2n isomer)*, C15:1, C16:0 (2n isomer)*, C16:2 (2n isomer)*) significantly increased following the extension of storage time in the control groups (CK-12h and CK-48h). However, seven representative LPCs in arginine-treated mushrooms were slightly decreased or showed no significant difference compared to the initial state with the extension of storage time (Figure 4C). Thus, these lipid-related metabolites may play important roles in arginine-induced softening inhibition in button mushroom.

### 3.5. Transcriptomic Differences Among Treatments

**Figure 5:**
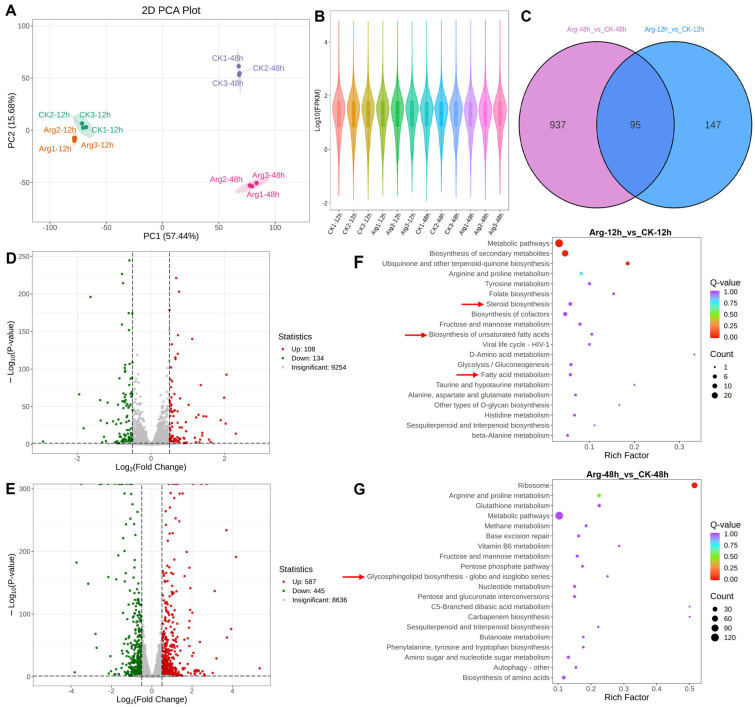
Overview of transcriptome analysis in control and arginine-treated mushrooms under refrigeration. (**A**) PCA of transcriptome data. (**B**) The violin plot displays an abundance of gene expression. (**C**) Venn diagram of differentially expressed genes (DEGs) in different groups. (**D**) The number of DEGs in Arg-12h vs. CK-12h. (**E**) The number of DEGs in Arg-48h vs. CK-48h. (**F**,**G**) KEGG enrichment of the DEGs in different groups (Arg-12h vs. CK-12h and Arg-48h vs. CK-48h), with red arrow highlighting the pathway related to lipid metabolism and metabolic pathways.

The transcriptome analyses were carried out to further investigate the changes in gene expression underlying arginine-delaying postharvest softening and senescence of mushrooms. Corresponding to metabolome analysis, 12 cDNA libraries (CK-12h, Arg-12h, CK-48h, Arg-48h), including three biological replications, were constructed and sequenced. PCA presented that all biological replicates were clustered together, and each group was clearly distinguished (Figure 5A). The violin plot indicated that the overall gene expression abundance was well, and their distribution in all groups was average (Figure 5B). As illustrated in the Venn diagram, a total of 4428 differentially expressed genes (DEGs, log2|FC| ≥ 1 and FDR < 0.05) were identified during the storage, and 1179 DEGs were detected in the mushroom of Arg-12h vs. CK-12h and Arg-48h vs. CK-48h, including 95 common DEGs and 1084 unique DEGs (Figure 5C). By comparing the two comparison groups (Arg-12h vs. CK-12h, and Arg-48h vs. CK-48h), 242 (108 up-regulated and 134 down-regulated), and 1032 (587 up-regulated and 445 down-regulated) DEGs were identified in the mushroom between Arg-12h vs. CK-12h, as well as Arg-48h vs. CK-48h, respectively (Figure 5D,E). KEGG analysis revealed that the DEGs of the two comparison groups (Arg-12h vs. CK-12h, and Arg-48h vs. CK-48h) were mainly enriched in the ‘metabolic pathways’, ‘steroid biosynthesis’, ‘biosynthesis of unsaturated fatty acids’, ‘fatty acid metabolism’, and ‘glycosphingolipid biosynthesis’ (Figure 5F,G), both of these are key pathways involved in lipid metabolism. These findings are consistent with the results from metabolome analysis, suggesting that lipid metabolism may be a major factor contributing to arginine-delaying postharvest ripening and senescence in mushrooms.

### 3.6. Construction of Gene Co-Expression Network

**Figure 6:**
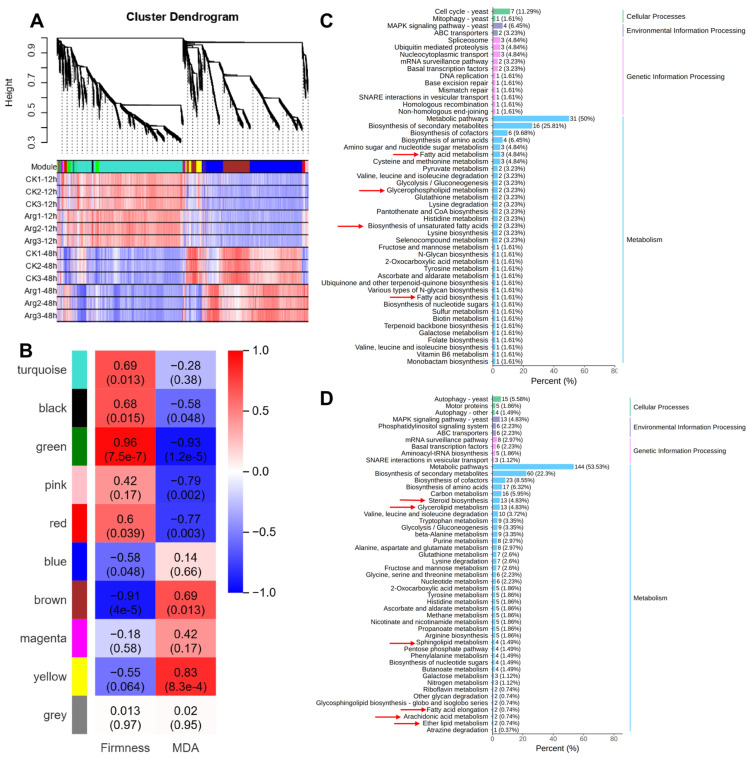
WGCNA in control and arginine-treated mushrooms during cold storage. (**A**) Hierarchical cluster tree. (**B**) Correlations between modules and physiological traits. (**C**) KEGG enrichment of the DEGs in the green module. (**D**) KEGG enrichment of the DEGs in the brown module, with red arrow highlighting the pathway related to lipid metabolism.

Further insight into the connection between lipid metabolism and arginine delay of button mushroom postharvest softening, WGCNA was used to investigate the co-expression networks of DEGs. The expression profiles of all filtered genes displayed high repeatability in each treatment, and a total of 10 co-expression modules were identified based on their similar expression patterns (Figure 6A). As depicted in Figure 6B, the firmness was significantly correlated (|r| > 0.90, *p* < 0.001) with the transcription of genes in the green and brown modules. Additionally, the expression of genes in the ‘green’ module exhibited a significant correlation with MDA content (Figure 6B). This implied that genes in these two modules might play a vital role in arginine-delaying postharvest ripening and senescence in mushrooms.

The enrichment pathways of candidate genes in green and brown modules were carried out by the KEGG analysis. The KEGG analysis displayed that the DEGs of the two modules were mainly enriched in metabolism, which primarily include amino acid metabolism, carbohydrate metabolism, and lipid metabolism. Among lipid metabolism, glycerolipid metabolism, biosynthesis of unsaturated fatty acids, glycerophospholipid metabolism, steroid biosynthesis, and fatty acid degradation (Figure 6C,D). These metabolic pathways might exert a vital function in the arginine-delayed postharvest softening of button mushrooms.

### 3.7. DEGs Analysis in Lipid Metabolism

**Figure 7:**
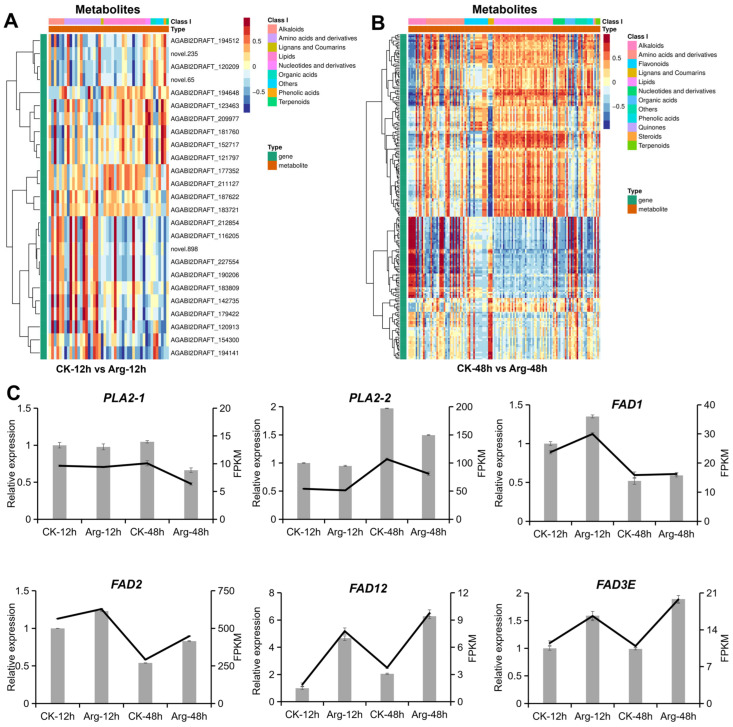
Correlation analysis of metabolome and transcriptome, and expression patterns of DEGs in lipid metabolism pathway. Heatmap of correlation between all upregulated and downregulated DEGs and DAMs in the comparison of CK-12h vs. Arg-12h (**A**) and CK-48h vs. Arg-48h (**B**). (**C**) qRT-PCR validation, the expression data (FPKM) obtained from RNA-seq are visualized using a line graph. Each value is expressed as mean ± standard error (n = 3).

Further insight into the correlation between DAMs and DEGs was gained by the correlation of all DAMs and DEGs in comparable groups, CK-12h vs. Arg-12h and CK-48h vs. Arg-48h, and DAMs and DEGs were found to be closely related to lipid metabolism (Figure 7A,B). Lipid metabolism was therefore proposed as a possible key factor in postharvest softening in mushrooms, and the related DAMs and DEGs were further analyzed. A total of 18 and 39 DEGs related to lipid metabolism were identified in comparable groups, CK-12h vs. Arg-12h and CK-48h vs. Arg-48h, respectively (Supplementary Table S2). Among these DEGs, the expression levels of eight genes (AGABI2DRAFT_212854, novel.898, AGABI2DRAFT_227554, AGABI2DRAFT_179422, AGABI2DRAFT_190206, novel.65, AGABI2DRAFT_116205, AGABI2DRAFT_194512) were markedly changed in the comparison of CK-12h vs. Arg-12h (Figure 7A, Table S3). In the comparison of CK-48h vs. Arg-48h, the expression levels of genes novel.65 and AGABI2DRAFT_194512 remained down-regulated, while another 19 genes significantly changed (Supplementary Table S4). These results once again confirm the involvement of lipid metabolism during the process of softening.

Among genes related to lipid metabolism, six DEGs related to biosynthesis of unsaturated fatty acids and fatty acid metabolism (*PLA2-1*, *PLA2-2*, *FAD1*, *FAD2*, *FAD12*, *FAD3E*) were selected for quantitative RT-PCR analysis to confirm the quality of RNA-seq data (Figure 7C). Results showed that the relative expression levels of *PLA2-1* and *PLA2-2* were significantly decreased in the arginine group. In contrast, the relative expression levels of the other five selected genes (*FAD1*, *FAD2*, *FAD12*, *FAD3E*) were significantly increased in the arginine group; these results were consistent with RNA-seq analysis.

## 4. Discussion

Button mushrooms have become a predominant choice for consumers in the market due to their rich nutritional value and pleasant taste. Its cultivation has developed rapidly, and it is the most widely cultivated mushroom globally. However, high respiration and transpiration rates make it difficult to preserve the quality of harvested mushrooms, resulting in the loss of taste and high nutritional value [28]. Although notable advancements have been made in browning and softening control technologies for mushrooms, our understanding of the specific mechanisms involved remains incomplete [29]. This knowledge gap impedes the development of safer and more efficient methods to control the softening and browning of mushrooms. Previous studies have demonstrated the excellent preservative effect of arginine on the postharvest freshness preservation of button mushroom [21], while the potential action mechanism of arginine requires further exploration to lay a foundation for the application of arginine and expand on its application in packaging systems, integration with cold-chain logistics, or compatibility with organic certification [30]. Therefore, we further studied the effect of arginine on the postharvest quality of button mushrooms and explored the underlying action mechanism.

Softening of edible mushrooms during postharvest storage is a key physiological process leading to senescence and a decrease in fruit body quality. This phenomenon is usually accompanied by browning and the production of a soft and spongy structure [31]. Compared with the values in the control group, the firmness increased by approximately 23.8% on the 8th day in the mushrooms of the 1.5 g L^−1^ arginine group, which serves as the primary indicator of structural preservation. A similar decline tendency was found by Lin et al., the firmness decreased with the storage duration; however, the phase-change materials and exogenous melatonin significantly maintained better textural attributes of button mushroom [32]. The softening of mushroom tissue is the result of several factors, including breakdown of the cell wall through metabolism and the active expression of carbohydrate-active enzymes. Polyphenol oxidase (PPO) is involved in browning, which is often associated with postharvest quality decline, though its direct role in softening may be indirect. Wang et al. found that ozone fumigation combined with polyethylene nano-packaging could delay the postharvest browning and softening of mushrooms by inhibiting the activity of PPO and chitinase [33]. Furthermore, the MDA content increases during senescence, accompanied by a rapid increase in membrane permeability, and a close connection between the tissue firmness and MDA content was proposed by Hu et al., who found that the sudden decline in firmness resulted from the increase in MDA content [34]. In this study, the decrease in MDA content under arginine treatment contributes to the maintenance of the firmness of button mushrooms and reflects the integrity of the cell wall. This enhanced firmness directly reflects the maintenance of cellular turgor and structural components, preventing the collapse of membrane systems that typically occurs during postharvest senescence.

In fruit and mushrooms, membrane lipid peroxidation increases. The MDA is considered the product of lipid peroxidation; its accumulation reflects the reduction in membrane integrity and the damage in cell wall structure, resulting in increased membrane leakage and increased cell senescence level [35]. In this study, a significant reduction in MDA content, a well-established biomarker of lipid peroxidation, indicates effective suppression of oxidative damage to membrane lipids. Similar results were found in the study of Li et al. [21]. Superoxide dismutase (SOD) plays an important role in maintaining the freshness of fruits and vegetables during storage because it can catalyze the dismutation of superoxide anions into hydrogen peroxide and oxygen, thus reducing oxidative stress, preventing cell damage, and extending the shelf life of produce [36]. Additionally, this reduction correlates with enhanced antioxidant enzyme activities, particularly SOD, suggesting that arginine treatment activates the mushroom’s endogenous defense systems against reactive oxygen species. Phenylalanine ammonium lyase (PAL), together with PPO, are the key enzymes in postharvest browning of fruits and mushrooms, since their processed products could lead to a limited shelf-life and negatively impact commercial value. The arginine application efficiently prevented the development of browning in mushrooms. Several reports also agreed that the coordinated action of enzymes such as SOD, PAL, and PPO responds to measures aiming at extending the shelf life in other mushrooms, table grapes, and litchi [37,38].

This study presents a novel approach by integrating metabolomic and transcriptomic analyses to elucidate the mechanism through which arginine alleviates postharvest senescence in button mushrooms. The utilization of high-throughput methods, such as metabolome and transcriptome analysis, allows for the investigation of global changes in metabolites and genes [39]. The integration of these approaches has proven powerful in gaining a deeper understanding of postharvest softening and browning in various mushrooms. Lipid metabolites associated with cell membrane degradation were predominantly up-regulated during ambient storage [28]. From metabolomic analysis, the observed changes in fatty acid composition provide the molecular basis for membrane stability. The increased levels of unsaturated fatty acids (UFAs) enhance membrane fluidity and flexibility, which are essential for maintaining membrane integrity under various stress conditions. Zhang et al. have demonstrated that membrane lipid metabolism responded to postharvest senescence delay, and alterations in the composition and content of UFAs and SFAs within membrane lipids affect the integrity and fluidity of fruit membranes, which are intimately connected with the chain length and unsaturation of fatty acids [40]. Dobón-Suárez et al. showed that preharvest applications of salicylic acid improved postharvest quality and enhanced chilling tolerance of green pepper fruit and resulted in the highest UFA/SFA ratio [41]. Also in green bell pepper methyl jasmonate treatment alleviates chilling injury by regulating membrane lipid composition [42]. To our knowledge, there are no studies to date that indicate the effect of arginine on the composition of fatty acids in button mushrooms. Treatment with arginine that helps preserve or increase the levels of UFAs and maintain a higher UFAs/SFAs ratio is thus crucial in mitigating softening and senescence in mushrooms. In addition, arginine application has also been shown to influence the content of lysophosphatidylethanolamines (LPEs) and lysophosphatidylcholines (LPCs). LPE, a natural phospholipid, has been investigated for retarding senescence and promoting the shelf life of fruit and other plant tissues, banana and tomato fruit. LPE has been found to inhibit the activity of phospholipase D (PLD), which is known to be activated during ethylene-induced senescence, and this activation leads to membrane breakdown [43]. LPCs are increasingly recognized as key markers/factors positively associated with cell damage, and lysoPC 18:1 could induce cell death in tobacco plants [44]. These results of both LPE and LPC alteration in response to arginine treatment provide an exact explanation of its positive effect. The regulatory effects of arginine on lipid metabolism observed in this study suggest potential transferability to other mushroom cultivars and even plant-based perishable commodities that experience similar lipid oxidation-induced quality deterioration. This cross-species applicability highlights the broad potential of arginine-based treatments in postharvest preservation strategies.

Phospholipases A2 (PLA2) are the primary enzymes that release fatty acids and lysophospholipids by hydrolyzing the *sn*-2 position of glycerophospholipids [45]. In this study, transcriptomic analysis reveals that arginine treatment upregulates fatty acid desaturase genes while downregulating PLA2 (*PLA2-1* (AGABI2DRAFT_120522) and *PLA2-2* (AGABI2DRAFT_64631), creating a favorable metabolic environment for unsaturated fatty acid accumulation. This genetic reprogramming ensures the maintenance of optimal membrane fluidity, preventing the phase transitions and increased rigidity that typically accompany the senescence of button mushrooms. These changes were similar to the changes in gene expression in blueberries after melatonin treatment [20]. A set of fatty acid desaturases (FADs) was also involved in the fatty acid desaturation in both phospholipid and galactolipid metabolism pathways, which could regulate the level of membrane lipid saturation. In many horticultural plants, the expression levels of the FAD genes have been shown to be positively correlated with stress resistance [46,47]. In this study, the content of UFAs was increased by arginine application, which promoted the conversion of SFAs to monounsaturated and polyunsaturated fatty acids through up-regulating the expression of key genes in FAD families (FAD12, Δ(12) fatty acid desaturase; FAD3E, Acyl-lipid omega-3 desaturase). The upregulation of key genes in the FAD gene families (*FAD1*, AGABI2DRAFT_187203; *FAD2*, AGABI2DRAFT_194591; *FAD12*, AGABI2DRAFT_187622; *FAD3E*, AGABI2DRAFT_226540) increased the content of UFA, which in turn alleviated the postharvest softening of mushrooms. In conclusion, arginine treatment stimulated lipid metabolism by down-regulating the gene expression of *PLA2s* and up-regulating the gene expression of *FADs*, promoting the formation of fatty acids and maintaining a higher level of UFAs, which enhanced the tolerance and delayed the senescence.

Metabolomic profiling identified significant alterations in lipid metabolites, particularly increased unsaturated fatty acids, while transcriptomic analyses revealed corresponding upregulation of fatty acid desaturase genes and downregulation of PLA2 genes. This dual-platform integration demonstrates how arginine orchestrates a comprehensive defense network by linking specific gene expression patterns with metabolic outcomes to maintain membrane integrity and delay senescence. The synergistic application of these complementary omics technologies represents a significant methodological advancement over previous studies that typically employed single-platform analyses, offering a more holistic understanding of the complex regulatory mechanisms underlying arginine-induced preservation in postharvest mushrooms. This study suggests that arginine may delay postharvest softening of button mushrooms by modulating lipid metabolism. The specific genes and molecular interactions involved in lipid metabolism should be further investigated through gene knockdown and other functional validation methods to better target regulatory mechanisms and enhance postharvest preservation in *A. bisporus*.

## 5. Conclusions

In summary, arginine treatment effectively maintained firmness and delayed senescence in button mushrooms. The application of arginine reduced MDA content and suppressed the activities of the enzyme PAL and PPO, while enhancing SOD activity. Metabolomic analysis revealed significant alteration in lipid-related metabolites, particularly free fatty acids, lysophosphatidylcholine (LPC), lysophosphatidylethanolamine (LPE), and phosphatidylcholine (PC). Notably, arginine treatment increased the levels of unsaturated fatty acids (UFAs), suggesting that lipid-related metabolites may play crucial roles in arginine-mediated inhibition of softening in button mushrooms. Transcriptomic analysis further demonstrated that DEGs were predominantly enriched in the lipid metabolism pathways, with key DEGs involved in lipid metabolism—specifically *PLA2* and *FAD*, exhibiting significant changes. The practical advantages of arginine, including its low cost, safety profile, and scalability potential, underscore its promise for commercial postharvest applications. Our findings demonstrate that arginine treatment orchestrates a coordinated response across physiological, biochemical, and molecular levels to maintain membrane integrity and significantly delay the senescence process in button mushrooms, offering promising applications of arginine for sustainable postharvest preservation strategies in the mushroom industry and potentially other perishable commodities.

## Data Availability

The original contributions presented in this study are included in the article/Supplementary Materials. Further inquiries can be directed to the corresponding authors.

## Supplementary Materials

The following supporting information can be downloaded at: https://www.mdpi.com/article/10.3390/foods14244359/s1, Table S1: Differential accumulation of lipid compounds in button mushroom in the comparison of CK-48h vs. Arg-48h, Table S2: DEGs related to lipid metabolism in the button mushroom, Table S3: DEGs related to lipid metabolism in the comparison of CK-12h vs. Arg-12h, Table S4: DEGs related to lipid metabolism in the comparison of CK-48h vs. Arg-48h.
