# Supplementary material for: Arginine Delays Postharvest Softening of Button Mushroom (Agaricus bisporus) via Lipid Metabolism Regulation
Source: Foods. 2025 Dec 18;14(24):4359. doi: 10.3390/foods14244359 (PMC12732925; doi:10.3390/foods14244359)
Supplement: Supplementary file 1 [file foods-14-04359-s001.zip › foods-3969164-supplementary.pdf]

Supplementary material

Table S1 Differential accumulation of lipid compounds in button mushroom in the comparison of CK-48h vs Arg-48h

Compounds	Class	CK1-48h	CK2-48h	CK3-48h	Arg1-48h	Arg2-48h	Arg3-48h	Fold_Change	Log2FC	Type
(E)-Linalool-1-oic acid	Free fatty acids	24332	30384	25015	58262	68822	59070	2.334775683	1.223283948	up
Myristic Acid	Free fatty acids	2106600	2711300	2558300	3867200	4913300	3978500	1.729752447	0.790565582	up
Pentadecanoic Acid	Free fatty acids	21946	32217	30662	61427	79740	67376	2.458508694	1.297783457	up
Palmitic acid	Free fatty acids	3943.7	6867.4	4990.2	11657	11885	10769	2.171403619	1.118627918	up
LysoPC 20:5	LPC	18126	20950	13241	37514	38614	41174	2.242139266	1.164875891	up
10-Heptadecenoic Acid	Free fatty acids	257900	216150	207340	619640	647360	618880	2.767695446	1.468685199	up
α-Linolenic Acid	Free fatty acids	3111300	3877000	3453200	5275800	6588900	5423500	1.655719964	0.727458687	up
γ-Linolenic Acid	Free fatty acids	21528000	25359000	23127000	34646000	40557000	36143000	1.590339075	0.669334394	up
Punicic acid (9Z,11E,13Z-octadecatrienoic acid)	Free fatty acids	1239400	1209500	1196600	4953700	4368000	4806500	3.875517762	1.954389065	up
11-Octadecanoic acid(Vaccenic acid)	Free fatty acids	289130	593620	351700	1644400	1822700	2156300	4.55538904	2.187574268	up
Elaidic Acid	Free fatty acids	1322800	2407000	1613600	6467300	6525200	7455100	3.8267021	1.936101595	up
4-Oxo-9Z,11Z,13E,15E-Octadecatetraenoic Acid	Free fatty acids	8453.8	10914	11049	15648	16714	16859	1.618217564	0.694405586	up
9-Oxo-10E,12Z-octadecadienoic acid	Free fatty acids	15058	18747	18992	65479	71429	68539	3.891262761	1.960238403	up
9-Hydroxy-10,12,15-octadecatrienoic acid	Free fatty acids	13141	12854	14303	47618	54589	54868	3.897836121	1.962673436	up
9(10)-EpOME;(9R,10S)-(12Z)-9,10-Epoxyoctadece noic acid	Free fatty acids	60482	69209	65265	492350	558610	502250	7.966977164	2.99403244	up
13(S)-HODE;13(S)-Hydroxyoctadeca-9Z,11E-dieno ic acid	Free fatty acids	225250	239460	240840	847160	940050	927610	3.847806676	1.944036316	up
12,13-Epoxy-9-Octadecenoic Acid	Free fatty acids	57339	58967	61222	476590	548060	536140	8.791796224	3.136157948	up
9S-Hydroxy-10E,12Z-octadecadienoic acid	Free fatty acids	230670	226540	231390	868570	939170	888460	3.915480685	1.969189431	up
Eicosadienoic acid	Free fatty acids	25831	42641	24074	165660	138310	192290	5.362306313	2.422853633	up
13S-Hydroperoxy-6Z,9Z,11E-octadecatrienoic acid	Free fatty acids	9748.4	13960	13694	30509	24976	25174	2.156519368	1.108704673	up
Eicosenoic acid	Free fatty acids	35768	58874	56707	297030	352650	383460	6.826209621	2.771084718	up
13S-Hydroperoxy-9Z,11E-octadecadienoic acid	Free fatty acids	255360	231650	222200	823120	804070	830830	3.465856375	1.793211871	up
9-Hydroxy-13-oxo-10-octadecenoic Acid	Free fatty acids	845940	769810	756590	1505700	1455900	1474700	1.870010201	0.90304614	up
9-Hydroxy-12-oxo-15(Z)-octadecenoic acid	Free fatty acids	84027	83851	93802	355480	403480	350600	4.24014063	2.084112114	up
9,12,13-Trihydroxy-10,15-octadecadienoic acid	Free fatty acids	698410	755140	745760	3925900	4000500	4316300	5.566609528	2.476798888	up
9,10,11-Trihydroxy-12-octadecenoic acid	Free fatty acids	348460	374520	361010	1748700	1910600	2038900	5.25669056	2.394154812	up
9,10,13-Trihydroxy-11-Octadecenoic Acid	Free fatty acids	5758400	6118100	6116600	29603000	31383000	31298000	5.12885495	2.358636771	up

1-α-Linolenoyl-glycerol*	Glycerol ester	234000	225620	213160	385090	360300	340100	1.613439757	0.690139712	up
1-Linoleoylglycerol*	Glycerol ester	70636	69085	73541	176310	180980	167210	2.459416117	1.29831585	up
LysoPG 16:1	Glycerol ester	9078.2	8548.9	9885.1	52260	57846	49043	5.784670074	2.532234678	up
LysoPE 20:2(2n isomer)*	LPE	5777	6035.9	5879.1	10047	12507	12350	1.972869093	0.980295231	up
N-(2-Hydroxyethyl)eicosapentaenoic acid	Free fatty acids	47595	47636	61300	89316	100320	87396	1.769821952	0.823604229	up
LysoPE 20:2	LPE	7643.6	8692.1	7389	13411	12299	13454	1.65076903	0.723138277	up
LysoPE 22:5	LPE	3429.1	4783.5	3324.6	8309.7	6210.6	6971.5	1.862826336	0.897493183	up
LysoPE 16:1(2n isomer)*	LPE	150440	117620	125970	1345300	1218900	1376500	10.00101515	3.322074543	up
LysoPE 16:1	LPE	118300	121880	121710	1299600	1150600	1351800	10.50595485	3.393135384	up
LysoPE 17:1	LPE	33895	29948	31964	190680	184330	171610	5.705428622	2.512335274	up
LysoPE 17:1(2n isomer)*	LPE	26179	30785	33587	198660	182710	164580	6.029199015	2.591966351	up

Table S2 DEGs related to lipid metabolism in the button mushroom

Treatments	geneID	metaID	Compounds
CK-12h_vs_Arg-12h	AGABI2DRAFT_212854	MW0016024	Azelaoyl PAF
	novel.898	MW0016024	Azelaoyl PAF
	AGABI2DRAFT_227554	MW0016024	Azelaoyl PAF
	AGABI2DRAFT_179422	MW0016024	Azelaoyl PAF
	AGABI2DRAFT_190206	MW0016024	Azelaoyl PAF
	AGABI2DRAFT_116205	MW0016024	Azelaoyl PAF
	AGABI2DRAFT_212854	Lmhp007598	LysoPC 19:2(2n isomer)
	AGABI2DRAFT_227554	Lmhp007598	LysoPC 19:2(2n isomer)
	AGABI2DRAFT_179422	Lmhp007598	LysoPC 19:2(2n isomer)
	AGABI2DRAFT_190206	Lmhp007598	LysoPC 19:2(2n isomer)
	novel.65	Lmhp007598	LysoPC 19:2(2n isomer)
	AGABI2DRAFT_116205	Lmhp007598	LysoPC 19:2(2n isomer)
	AGABI2DRAFT_194512	Lmhp007598	LysoPC 19:2(2n isomer)
	AGABI2DRAFT_212854	Lmhp012042	2-Linoleoylglycerol*
	novel.898	Lmhp012042	2-Linoleoylglycerol*
	AGABI2DRAFT_227554	Lmhp012042	2-Linoleoylglycerol*
	AGABI2DRAFT_190206	Lmhp012042	2-Linoleoylglycerol*
	AGABI2DRAFT_116205	Lmhp012042	2-Linoleoylglycerol*
CK-48h_vs_Arg-48h	AGABI2DRAFT_187489	Lmhp009129	LysoPC 15:0(2n isomer)
	AGABI2DRAFT_198988	Lmhp009129	LysoPC 15:0(2n isomer)
	AGABI2DRAFT_192211	Lmhp009129	LysoPC 15:0(2n isomer)
	novel.413	Lmhp009129	LysoPC 15:0(2n isomer)
	AGABI2DRAFT_65743	Lmhp009129	LysoPC 15:0(2n isomer)
	novel.506	Lmhp009129	LysoPC 15:0(2n isomer)
	AGABI2DRAFT_180624	Lmhp009129	LysoPC 15:0(2n isomer)
	AGABI2DRAFT_204597	Lmhp009129	LysoPC 15:0(2n isomer)
	novel.492	Lmhp009129	LysoPC 15:0(2n isomer)
	novel.628	Lmhp009129	LysoPC 15:0(2n isomer)
	AGABI2DRAFT_195080	Lmhp009129	LysoPC 15:0(2n isomer)
	AGABI2DRAFT_198988	Lmhp007840	LysoPC 19:2
	AGABI2DRAFT_120209	Lmhp007840	LysoPC 19:2
	AGABI2DRAFT_65743	Lmhp007840	LysoPC 19:2

AGABI2DRAFT_139988	Lmhp007840	LysoPC 19:2
AGABI2DRAFT_191413	Lmhp007840	LysoPC 19:2
AGABI2DRAFT_204597	Lmhp007840	LysoPC 19:2
AGABI2DRAFT_181085	Lmhp007598	LysoPC 19:2(2n isomer)
AGABI2DRAFT_194512	Lmhp007598	LysoPC 19:2(2n isomer)
novel.65	Lmhp007598	LysoPC 19:2(2n isomer)
novel.628	pmd0130	LysoPC 14:0
AGABI2DRAFT_209045	pmd0146	LysoPC 20:2(2n isomer)
AGABI2DRAFT_193833	pmd0146	LysoPC 20:2(2n isomer)
AGABI2DRAFT_209045	pmb0863	LysoPC 16:2(2n isomer)
AGABI2DRAFT_193833	pmb0863	LysoPC 16:2(2n isomer)
AGABI2DRAFT_228161	pmb0863	LysoPC 16:2(2n isomer)
AGABI2DRAFT_194638	pmb0863	LysoPC 16:2(2n isomer)
AGABI2DRAFT_209045	pmb0865	LysoPC 18:3(2n isomer)
AGABI2DRAFT_209045	Hmqp006235	LysoPC 18:4
AGABI2DRAFT_193833	Hmqp006235	LysoPC 18:4
AGABI2DRAFT_228161	Hmqp006235	LysoPC 18:4
AGABI2DRAFT_206562	Hmqp006235	LysoPC 18:4
AGABI2DRAFT_209045	Lmhp008833	LysoPC 16:1(2n isomer)
AGABI2DRAFT_193833	Lmhp008833	LysoPC 16:1(2n isomer)
AGABI2DRAFT_194638	Lmhp008833	LysoPC 16:1(2n isomer)
AGABI2DRAFT_209045	Lmhp011549	LysoPC 20:1
AGABI2DRAFT_193833	Lmhp011549	LysoPC 20:1
AGABI2DRAFT_194638	Lmhp011549	LysoPC 20:1
AGABI2DRAFT_195080	Lmhp010190	LysoPC 18:1(2n isomer)

Table S3 DEGs related to lipid metabolism in the comparison of CK-12h vs Arg-12h

Index	CK1-12h	CK2-12h	CK3-12h	Arg1-12h	Arg2-12h	Arg3-12h	Log2FC	Regulation
AGABI2DRAFT_194141	22.0128	18.9239	21.956	42.3405	43.2328	43.4991	1.026660532	up
AGABI2DRAFT_194648	21.4338	22.8692	22.0067	10.7695	11.0021	10.9815	-1.028395215	down
AGABI2DRAFT_154300	63.3365	61.8436	59.5624	131.8326	140.5213	133.1378	1.123303223	up
AGABI2DRAFT_142735	5.5561	5.6286	5.8317	22.5998	23.3444	25.2044	2.053155692	up
**AGABI2DRAFT_212854**	**15.4815**	**14.319**	**16.1162**	**38.4329**	**39.7312**	**40.1222**	**1.35456195**	**up**
**novel.898**	**6.0426**	**6.5322**	**7.2878**	**16.6919**	**16.5594**	**15.8192**	**1.294447402**	**up**
AGABI2DRAFT_183809	14.4929	15.6077	14.1476	41.5423	43.0136	35.5582	1.430032531	up
**AGABI2DRAFT_227554**	**3.2894**	**3.5296**	**2.7081**	**8.9367**	**9.1697**	**9.0844**	**1.501962907**	**up**
**AGABI2DRAFT_179422**	**17.752**	**19.0012**	**15.0162**	**37.8536**	**45.3007**	**39.3754**	**1.23155953**	**up**
**AGABI2DRAFT_190206**	**4.4363**	**4.4342**	**3.0046**	**15.0209**	**14.9646**	**14.5796**	**1.896861093**	**up**
**novel.65**	**42.4179**	**45.0922**	**43.0239**	**19.7471**	**18.7397**	**18.9248**	**-1.195290165**	**down**
**AGABI2DRAFT_116205**	**2.8734**	**3.2975**	**1.9859**	**6.7614**	**6.2572**	**5.7959**	**1.195055325**	**up**
AGABI2DRAFT_120913	5.8508	5.8481	5.956	22.8809	25.8039	22.0259	1.990921475	up
AGABI2DRAFT_187622	2.1858	1.9277	1.5563	8.0338	7.0961	8.2097	2.030779093	up
**AGABI2DRAFT_194512**	**18.7321**	**18.134**	**19.2919**	**7.7953**	**7.1599**	**6.83**	**-1.375901159**	**down**
AGABI2DRAFT_152717	382.0575	400.4121	405.1875	122.0316	140.8954	121.1758	-1.639819617	down
AGABI2DRAFT_181760	54.4651	55.0476	62.2977	15.8865	14.7538	14.1554	-1.949174077	down
AGABI2DRAFT_121797	141.2128	140.4419	139.2382	44.2231	64.0981	56.6855	-1.363745663	down
AGABI2DRAFT_123463	9.8907	12.9568	12.3955	3.1737	3.595	3.2365	-1.828048059	down
AGABI2DRAFT_209977	95.8463	17.5766	62.3751	9.2297	3.0904	10.887	-2.929165642	down

Table S4 DEGs related to lipid metabolism in the comparison of CK-48h vs Arg-48h

Index	CK1-48h	CK2-48h	CK3-48h	Arg1-48h	Arg2-48h	Arg3-48h	Log2FC	Regulation
AGABI2DRAFT_175622	536.265	502.93	511.1775	235.7618	236.8578	238.3413	-1.105637128	down
**AGABI2DRAFT_187489**	**138.1404**	**142.0484**	**140.0813**	**45.1233**	**46.1004**	**48.4994**	**-1.569703642**	**down**
AGABI2DRAFT_192200	486.7602	450.3734	442.6725	39.3331	41.2337	40.4617	-3.492354039	down
AGABI2DRAFT_192951	294.9329	302.0332	306.9887	609.0925	598.1835	607.1898	1.024669522	up
AGABI2DRAFT_193061	3382.8411	3247.2122	3245.4581	1365.4082	1365.5969	1368.8404	-1.24904172	down
**AGABI2DRAFT_198988**	**248.9715**	**248.5539**	**246.5422**	**88.6003**	**88.6115**	**85.8059**	**-1.480768736**	**down**
AGABI2DRAFT_239305	320.5329	329.7687	305.766	68.7374	71.6498	69.7362	-2.166624123	down
AGABI2DRAFT_241547	407.5273	410.3573	392.9528	69.8293	66.8366	61.9889	-2.587397451	down
AGABI2DRAFT_60873	1103.9422	1094.8562	1083.4277	534.488	545.3135	523.5362	-1.014186828	down
AGABI2DRAFT_77253	948.0356	920.5904	921.0888	165.6952	165.3938	154.9604	-2.501342157	down
**AGABI2DRAFT_120209**	**1214.0262**	**1199.4016**	**1211.9158**	**469.372**	**480.309**	**448.049**	**-1.355575656**	**down**
AGABI2DRAFT_135027	329.411	319.3409	318.4232	157.5579	156.8351	155.3447	-1.022604986	down
novel.177	111.298	115.8878	107.9375	41.1535	40.5672	42.0254	-1.417789312	down
**AGABI2DRAFT_192211**	**87.1131**	**83.9694**	**82.2262**	**32.0839**	**31.9095**	**33.824**	**-1.353783129**	**down**
**novel.413**	**35.4605**	**35.2304**	**34.6683**	**11.0824**	**10.583**	**11.233**	**-1.659747459**	**down**
AGABI2DRAFT_145743	109.1333	102.1234	106.0288	22.844	24.1421	23.3568	-2.154737214	down
**AGABI2DRAFT_65743**	**78.599**	**76.6959**	**74.6473**	**22.3364**	**22.7548**	**21.7289**	**-1.763609798**	**down**
AGABI2DRAFT_208623	92.618	92.184	93.1066	38.0594	34.6702	35.5997	-1.339143344	down
**AGABI2DRAFT_139988**	**118.9539**	**112.1637**	**117.3828**	**11.3603**	**13.1677**	**14.2757**	**-3.150096231**	**down**
**AGABI2DRAFT_181085**	**42.2152**	**42.5773**	**44.9057**	**91.8453**	**93.8854**	**83.4397**	**1.073148937**	**up**
**novel.506**	**146.49**	**149.1068**	**146.2974**	**65.101**	**68.1385**	**63.1568**	**-1.150506063**	**down**
**AGABI2DRAFT_191413**	**688.7101**	**733.4299**	**748.9818**	**1588.2201**	**1601.1522**	**1487.662**	**1.126961204**	**up**
AGABI2DRAFT_203042	141.8025	143.806	143.3597	53.3074	55.531	47.9636	-1.43205471	down
**AGABI2DRAFT_180624**	**71.1466**	**69.1318**	**67.7042**	**32.1973**	**29.3227**	**32.6926**	**-1.122956371**	**down**
AGABI2DRAFT_239576	319.4686	314.103	268.461	137.7827	127.2759	135.572	-1.151277465	down
AGABI2DRAFT_183321	9.2964	9.4296	8.8801	20.7758	19.7124	21.4224	1.184737549	up
**AGABI2DRAFT_194512**	**23.8608**	**24.1548**	**23.1688**	**9.7014**	**8.7382**	**10.3685**	**-1.285551415**	**down**
AGABI2DRAFT_193931	130.5456	150.1888	145.6372	314.4369	321.9231	293.957	1.145710317	up
AGABI2DRAFT_180292	71.8242	60.9299	63.6375	24.9207	26.4093	23.5677	-1.372139833	down
AGABI2DRAFT_195858	14.3513	14.3877	15.4151	35.032	34.9825	34.1043	1.25689681	up
**AGABI2DRAFT_204597**	**25.353**	**25.3838**	**23.908**	**12.6865**	**11.6752**	**12.3437**	**-1.004148379**	**down**

AGABI2DRAFT_143474	19.5178	18.0438	18.1934	6.3693	6.2646	7.1322	-1.477677274	down
AGABI2DRAFT_196111	41.4318	38.0241	41.3211	18.8521	14.8955	16.6594	-1.240192035	down
AGABI2DRAFT_206844	63.9839	69.4979	66.3522	28.1577	27.9071	26.9254	-1.247661372	down
novel.235	16.0036	14.9983	18.0089	3.9463	3.1147	4.5643	-2.056631944	down
**novel.65**	**65.2664**	**74.8212**	**71.502**	**32.4288**	**37.5635**	**31.8058**	**-1.035965235**	**down**
AGABI2DRAFT_207558	5.492	6.2135	6.6533	12.2778	13.3372	12.5898	1.076942903	up
AGABI2DRAFT_192245	15.5083	13.2507	11.6944	4.0186	3.7759	3.8983	-1.771494617	down
AGABI2DRAFT_138064	6.0499	5.079	6.4825	0.8875	1.2452	2.1633	-2.023318303	down
AGABI2DRAFT_137582	13.5542	12.0833	14.5824	6.3943	4.9845	6.1852	-1.175499433	down
**novel.492**	**7.8379**	**7.9436**	**8.3536**	**2.3904**	**3.0836**	**4.8333**	**-1.21258801**	**down**
AGABI2DRAFT_194715	5.4276	3.2401	2.2867	0.247	0.3898	0.129	-3.82306222	down
AGABI2DRAFT_180530	1.2443	1.9521	2.7898	0.2363	0.6839	0.432	-2.133282331	down
**novel.628**	**3.557**	**4.2065**	**3.4939**	**0.7769**	**1.4015**	**1.3913**	**-1.642056456**	**down**
AGABI2DRAFT_139832	1.8226	2.0222	3.0995	0.7238	0.5712	0.6616	-1.806494168	down
AGABI2DRAFT_188058	23.2641	21.4607	23.972	11.4912	9.2517	13.0893	-1.002996602	down
AGABI2DRAFT_193529	1095.9282	1087.2177	1090.5743	314.8424	312.6999	294.7119	-1.808024962	down
AGABI2DRAFT_189525	85.5615	89.859	87.7692	225.6952	232.8314	220.3424	1.386785225	up
AGABI2DRAFT_195178	103.6666	102.3571	107.4934	210.78	208.5724	212.555	1.030328856	up
AGABI2DRAFT_66531	16.4168	16.6126	19.8811	7.4352	8.9944	7.4044	-1.132141163	down
AGABI2DRAFT_181542	0.6222	0.5079	0.6291	1.1498	1.21	1.2012	1.034326198	up
AGABI2DRAFT_222775	29.7377	27.8121	31.7327	63.9851	65.3513	61.8074	1.116980561	up
AGABI2DRAFT_194647	727.5879	782.3033	799.2727	1824.9097	1795.1224	1832.6371	1.259136628	up
AGABI2DRAFT_116453	524.5287	551.3734	538.3282	1052.4608	998.6202	1147.4921	1.005689897	up
AGABI2DRAFT_191521	736.5648	743.0061	729.3463	1608.7695	1578.0111	1627.9534	1.143429674	up
AGABI2DRAFT_200333	77.1375	79.1943	75.8309	178.1719	171.6047	166.8421	1.173770998	up
AGABI2DRAFT_69119	478.6831	476.788	469.3936	219.4404	225.4116	229.7479	-1.059672064	down
AGABI2DRAFT_190959	16.3811	15.7105	17.2888	48.5285	50.9699	51.4819	1.630757344	up
AGABI2DRAFT_188981	20.9397	22.4573	19.2279	42.8067	43.5253	41.589	1.050681117	up
AGABI2DRAFT_183705	32.4539	30.8907	29.552	65.5949	60.0995	70.5026	1.097474943	up
AGABI2DRAFT_191998	26.2114	28.5132	26.7073	59.5596	61.287	51.7544	1.10435263	up
AGABI2DRAFT_117546	6.6196	7.341	7.4454	19.4529	19.2746	17.7764	1.420810194	up
AGABI2DRAFT_72455	0.9609	1.3597	1.1335	10.8427	10.3981	10.2309	3.211561395	up
AGABI2DRAFT_145709	7.4097	7.7744	8.5352	3.6091	3.7254	3.266	-1.142118346	down
AGABI2DRAFT_193447	32.8929	28.0189	33.6969	14.6517	15.1699	16.2937	-1.019198355	down
AGABI2DRAFT_213173	1.4658	1.8335	1.6939	0.1715	0.3008	0.8959	-1.857101001	down

AGABI2DRAFT_178627	1.8438	2.4063	2.1918	3.9181	4.6958	4.4058	1.035602856	up
AGABI2DRAFT_75872	303.4821	283.8278	239.8285	1200.1889	880.966	1933.7613	2.295308161	up
AGABI2DRAFT_75975	1811.2927	1620.3701	1459.0217	6087.5873	5030.5569	10933.8141	2.188620492	up
AGABI2DRAFT_196285	155.6874	159.1327	129.2866	659.1293	464.9075	1101.3286	2.341080254	up
**AGABI2DRAFT_195080**	**22.2652**	**20.7717**	**19.4985**	**115.953**	**105.4988**	**119.8164**	**2.467018696**	**up**
AGABI2DRAFT_196147	1245.4128	1217.6893	1198.1477	2889.034	2761.3105	2841.3201	1.232996736	up
AGABI2DRAFT_194648	2.8689	3.0035	3.0968	26.4602	24.1029	26.5366	3.123391841	up
AGABI2DRAFT_175783	296.9798	304.604	310.5174	108.5013	107.9144	113.0482	-1.449823732	down
AGABI2DRAFT_132801	303.1727	280.6746	280.1858	128.7518	117.6244	119.9077	-1.218436502	down
AGABI2DRAFT_195900	10.6131	10.9514	9.9474	24.2307	25.0997	25.6792	1.270650204	up
AGABI2DRAFT_137782	165.1993	161.0359	147.4715	341.2052	306.8498	319.3314	1.049945249	up
AGABI2DRAFT_217766	8.0432	7.6425	6.8997	2.5685	3.2368	2.3851	-1.444973218	down
novel.475	1.92	2.1677	1.8174	6.7807	6.4315	6.8972	1.788814927	up
AGABI2DRAFT_43781	14.3213	12.6373	12.447	27.6764	25.2418	25.1495	1.005500586	up
AGABI2DRAFT_46023	51.4146	52.8538	53.0068	24.2202	23.4026	26.9123	-1.058224387	down
AGABI2DRAFT_153035	2.165	2.7013	2.1596	0.9904	0.8742	1.2016	-1.175899019	down
novel.116	7.2295	8.312	8.7314	15.5705	14.5393	18.7862	1.029685205	up
AGABI2DRAFT_183809	1.6195	1.7188	2.1547	26.4354	21.4521	24.0185	3.729918511	up
AGABI2DRAFT_142735	6.7419	7.4456	6.2287	16.2945	16.9114	15.8902	1.286504221	up
AGABI2DRAFT_195896	3.7536	4.371	5.3242	10.7032	10.904	9.0403	1.208774663	up
AGABI2DRAFT_137962	11.2387	12.2757	12.4007	23.3367	25.1711	24.8472	1.049740034	up
AGABI2DRAFT_75612	364.4875	333.899	241.9762	1234.6728	911.9736	1883.1979	2.115666957	up
AGABI2DRAFT_75878	503.7755	436.403	344.6059	1200.5069	1118.3465	2116.443	1.80348688	up
AGABI2DRAFT_67213	15.1562	13.4042	18.1985	38.9641	25.4005	32.0595	1.063827969	up
AGABI2DRAFT_123463	2.1175	2.3922	2.4864	0.8951	0.7065	0.6234	-1.629514782	down
AGABI2DRAFT_187933	297.8967	292.6123	301.3606	93.5283	96.3651	93.1279	-1.636719836	down
AGABI2DRAFT_207044	1444.9077	1284.7637	1277.1947	226.231	220.7963	223.907	-2.559106192	down
AGABI2DRAFT_63000	5744.5375	6104.3691	5985.685	12977.394	12852.8756	12087.6832	1.107989437	up
AGABI2DRAFT_212410	96.4483	95.1762	93.316	39.4779	38.4789	37.994	-1.277564822	down
AGABI2DRAFT_152717	159.3964	159.6694	143.9158	13.3898	9.1399	11.7196	-3.733799386	down
AGABI2DRAFT_79454	24.6174	26.6409	27.2214	96.7394	94.6262	91.5845	1.870292417	up
AGABI2DRAFT_123218	253.3106	261.5562	257.0575	106.0561	98.261	106.2037	-1.293973765	down
AGABI2DRAFT_210909	66.6876	63.4008	62.2693	29.013	28.2865	32.0042	-1.088278008	down
AGABI2DRAFT_119762	30.7161	29.6759	30.2015	12.6076	13.6327	12.8486	-1.193955963	down

AGABI2DRAFT_121797	91.0768	84.0212	77.1226	11.2463	11.0953	15.053	-2.736347214	down
AGABI2DRAFT_122919	17.2192	16.5401	17.2886	7.4687	8.1379	7.5607	-1.121144218	down
AGABI2DRAFT_116089	71.0215	65.8441	62.3203	31.053	34.5548	31.3537	-1.020107746	down
AGABI2DRAFT_144304	3.0852	4.0681	4.191	7.6232	9.4906	7.7504	1.15263338	up
novel.988	1.3994	1.2996	1.9457	5.7309	6.4329	5.3882	1.935337672	up
AGABI2DRAFT_177985	1.3508	2.1504	1.8211	0.4917	0.9487	1.0274	-1.092730874	down
AGABI2DRAFT_209045	11.6294	13.6064	13.4224	50.7369	40.8106	45.4963	1.846953239	up
**AGABI2DRAFT_193833**	**15.5045**	**15.2587**	**14.5377**	**33.6507**	**32.9114**	**30.9426**	**1.125728294**	**up**
AGABI2DRAFT_191438	39.5531	41.329	40.3644	114.4535	110.8147	122.3815	1.539027491	up
**AGABI2DRAFT_228161**	**3.4791**	**3.6456**	**4.3194**	**50.3049**	**45.296**	**51.4971**	**3.703004627**	**up**
AGABI2DRAFT_138958	28.471	27.3755	26.3619	89.1924	88.9296	91.8609	1.734515594	up
AGABI2DRAFT_211127	1.5835	1.5278	2.0742	30.8124	30.1506	31.8604	4.179194788	up
AGABI2DRAFT_194619	12.2676	13.5991	14.3584	48.5402	49.874	51.4755	1.917317164	up
AGABI2DRAFT_177352	15.7158	13.2066	13.7758	56.7	50.237	51.2781	1.908193005	up
AGABI2DRAFT_190737	130.9201	146.482	145.9268	281.8847	287.5499	267.5897	1.00363214	up
**AGABI2DRAFT_206562**	**0.792**	**0.804**	**0.6615**	**11.469**	**11.0801**	**11.5885**	**3.938978041**	**up**
AGABI2DRAFT_194017	12.5438	11.5281	12.2021	24.6017	23.1876	25.6837	1.036844854	up
AGABI2DRAFT_195591	8.0037	9.6822	8.8852	30.9884	30.8095	28.8609	1.792023419	up
AGABI2DRAFT_183903	7.1347	6.4284	6.1247	21.6886	17.8374	20.2322	1.621125545	up
AGABI2DRAFT_195217	4.6231	5.8532	4.9206	26.8371	26.7506	26.0004	2.392154297	up
AGABI2DRAFT_208395	2.1057	2.3524	2.5214	5.7488	7.7692	8.0286	1.644121393	up
AGABI2DRAFT_117541	1.8594	2.3467	1.6769	5.1039	5.3711	4.1279	1.334584641	up
AGABI2DRAFT_228258	2.2068	2.4675	2.7892	5.4223	4.3023	5.3049	1.030195978	up
**AGABI2DRAFT_194638**	**14.9341**	**14.9484**	**15.6468**	**35.9885**	**32.8612**	**34.9548**	**1.208543639**	**up**
AGABI2DRAFT_191557	0.1862	0.1976	0.1882	1.3554	1.2837	0.7788	2.601603147	up
AGABI2DRAFT_136353	397.9299	417.2167	413.0867	947.0968	970.6173	933.3838	1.234556837	up
AGABI2DRAFT_189342	138.4115	150.5449	152.1271	981.4948	962.3761	943.3069	2.73054276	up
AGABI2DRAFT_194914	92.0945	100.7277	95.1547	399.4507	396.2648	373.3832	2.041584303	up
AGABI2DRAFT_139842	40.7035	43.7375	40.5244	94.1942	94.4517	98.759	1.22131035	up
AGABI2DRAFT_193505	140.8796	132.614	134.7151	289.6949	270.178	273.1266	1.048317631	up
AGABI2DRAFT_188421	9.1777	9.1134	8.499	35.9219	33.3642	37.734	2.017482243	up
AGABI2DRAFT_239609	19.8395	19.1257	20.3874	39.8672	41.5361	41.1411	1.064648251	up
AGABI2DRAFT_239600	14.4259	17.3564	14.731	53.9677	54.0844	59.719	1.871569636	up
AGABI2DRAFT_188285	27.0817	29.0022	30.5855	57.0318	57.7583	60.5451	1.035846172	up
AGABI2DRAFT_139552	21.8917	19.3998	22.32	50.124	50.0223	50.5734	1.262668521	up

AGABI2DRAFT_195472	39.5438	39.9469	38.5544	79.0606	83.4491	76.6354	1.038082135	up
AGABI2DRAFT_180469	54.0159	48.3292	50.0171	22.6352	24.7586	25.3766	-1.048153637	down
AGABI2DRAFT_194192	7.8382	7.8639	7.6962	16.7614	16.8568	17.0214	1.133194355	up
AGABI2DRAFT_138563	7.4276	8.4667	8.623	31.0929	31.614	34.3649	2.005031266	up
AGABI2DRAFT_193080	33.237	34.2147	30.2769	17.6576	14.7967	14.2333	-1.044198275	down
AGABI2DRAFT_187568	2.9321	3.4293	2.4202	7.9069	8.8709	8.3967	1.540569606	up
AGABI2DRAFT_182285	5.6669	5.4503	5.1033	11.2165	10.4808	10.556	1.011330238	up
AGABI2DRAFT_188016	2.6674	2.3254	3.1467	6.2767	6.1308	5.3616	1.14481967	up
AGABI2DRAFT_43698	4.9137	5.5171	5.9045	10.6541	11.9483	13.7297	1.171700716	up
AGABI2DRAFT_119234	2.751	2.1897	2.0862	4.6731	5.8837	5.4049	1.20018885	up
AGABI2DRAFT_181511	0.7078	0.3219	0.7668	2.0981	1.9174	0.8075	1.441757247	up
novel.250	0.7147	0.6953	0.9033	1.4308	1.7795	2.378	1.287942918	up
AGABI2DRAFT_123111	0.3507	0.3722	0.5573	1.3132	0.8637	1.3147	1.466653818	up
novel.738	0.825	1.626	1.0725	2.9601	2.7087	2.9579	1.317886686	up
AGABI2DRAFT_192723	3.5996	4.6245	4.0867	15.7236	16.9821	18.2271	2.06965324	up
AGABI2DRAFT_115890	6.3939	6.5434	6.5641	13.5372	12.4088	12.9883	1.017378367	up
novel.994	4.8232	5.3514	8.2021	15.3224	14.1089	12.0052	1.193056463	up
AGABI2DRAFT_195510	1.4955	2.0406	2.0162	4.1216	3.1098	4.3058	1.076998931	up
AGABI2DRAFT_191084	0.1468	0.5453	0.2969	1.6832	1.0966	1.5072	2.149431353	up
novel.776	2.5841	2.1548	3.3594	7.0548	3.8181	5.4748	1.033208294	up
